# Biofeedback physical regulation of hypertension based on acupoints: A clinical trial

**DOI:** 10.1097/MD.0000000000033946

**Published:** 2023-06-23

**Authors:** Ling-Hui Ma, Zhou Zhang, Liang-Xiao Ma, Jie-Dan Mu, Xu Qian, Qin-Yong Zhang, Tian-Yi Sun

**Affiliations:** a School of Acupuncture-Moxibustion and Tuina, Beijing University of Chinese Medicine, Beijing, China; b The Key Unit of State Administration of Traditional Chinese Medicine, Evaluation of Characteristic Acupuncture Therapy, Beijing, China.

**Keywords:** body-mind holistic regulation, hypertension, randomized controlled trial, stimulation frequencies, study protocol, transcutaneous electrical acupoint stimulation

## Abstract

Hypertension is a predominant risk factor for cardiovascular disease and has imposed a global disease burden. Poor medication compliance is the major obstacle to antihypertensive drug therapy, and negative mood status is also detrimental to blood pressure (BP) management. While transcutaneous electrical acupoint stimulation (TEAS), as an electrical stimulation modality for biofeedback physical regulation based on acupoints, offers a such nondrug alternative option that is noninvasive, safe, and effective with high adherence. However, the optimal stimulation parameters of TEAS for hypertension remain unclear, especially the frequency, which needs further exploration.

**Objective:**

The study aims to investigate the efficacy of TEAS for hypertension, and to screen the optimal electrical stimulation frequency.

**Methods:**

This is an 8-week, randomized, controlled pilot trial with 3 parallel groups. In a ratio of 1:1:1, 120 patients with stage 1 hypertension will be divided into the TEAS-2Hz group, TEAS-10Hz group, or usual care group. All patients will receive the usual care for hypertension including lifestyle education, etc. Additionally, the 2 TEAS groups will receive 12 sessions of TEAS interventions at 2 Hz or 10 Hz, 3 times weekly for 30 minutes each, with 4 weeks of follow-up. The main outcome will be the change from baseline to week 4 in systolic BP among the groups. Secondary outcomes consist of changes in diastolic BP, mean arterial pressure, heart rate, heart rate variability, medication adherence, and quality of life. The safety outcomes will be any adverse event during the treatment.

**Discussion:**

As a pre-study for the next large clinical trial of TEAS for hypertension, this study will offer references for optimized frequency of biofeedback electrical devices and promote more consciousness of the benefits of body-mind holistic regulation of BP, thereby achieving proactive and overall process management of BP.

## 1. Introduction

Hypertension, the most prevalent chronic condition, is regarded as the leading risk factor for cardiovascular disease and renal diseases.^[1–3]^ In 2019, high systolic blood pressure (BP) was the dominant hazard factor for attributable mortality globally, accounting for 19.2% of total deaths.^[4]^ Hypertension with increasing prevalence imposes a heavy burden of diseases worldwide.^[5,6]^ Although long-term adherence to lifestyle interventions and antihypertensive medication are recommended by various guidelines for hypertension, fewer than 50% of those on treatment had achieved BP control.^[7–10]^ Difficulty in lifestyle changes and limitations in pharmacotherapy owing to adverse effects, expense, and compliance of patients are primarily responsible for uncontrolled BP.^[11,12]^ As a consequence, a wider range of antihypertensive interventions are increasingly required for the needs of distinct hypertensive groups.

Acupuncture, a vital component of traditional Chinese medicine, has been utilized as a nondrug therapy for treating patients with cardiovascular disease.^[13–15]^ Although the conclusions are varied, most studies suggest that acupuncture is effective in lowering BP as an adjunctive therapy.^[3,16–18]^ Transcutaneous electrical acupoint stimulation (TEAS), as the electrical stimulation portion of biofeedback physical regulation based on acupoints, is a novel acupuncture technique that is noninvasive, integrating the functions of modern transcutaneous electrical nerve stimulation (TENS) and conventional acupoint stimulations.^[19]^ By applying electro-stimulation pulses on aimed acupoints, TEAS delivers similar effects to electroacupuncture, but without the risks of invasive infection, patients psychological fears, or manipulator bias.^[20]^ Meanwhile, Along with the stimulation sites, the stimulation parameters are another crucial factor in the efficacy of TEAS. Evidence for a potential hypotensive effect of TEAS has been provided by some studies, but the optimal stimulation parameters remain unclear.^[21–23]^ The stimulation parameters of TEAS mainly consist of intensity, waveform, and frequency, among which the frequencies used vary widely in different studies, ranging from low frequency to high frequency.^[24,25]^ Studies find that both high frequency and low frequency electrical stimulation have shown some vasodilatory effects.^[26,27]^ but for decreasing BP, low frequency seems to perform better, which may also correlate with its modulation of autonomic nerves.^[21,28,29]^ Also, the low frequency of electrical stimulation is a wide range that requires to be selected optimally.

Based on the above, the pilot trial is expected to investigate the effectiveness and safety of TEAS for hypertension by comparing it with usual care; to explore the optimal transcutaneous electrical stimulation frequency by comparing different stimulation frequencies at 2 Hz and 10 Hz. Further, it will lay a foundation for future large clinical trials and provide a reference for the parameter setting of biofeedback electric stimulation devices.

## 2. Methods

### 2.1. Study design

**Figure 1.**
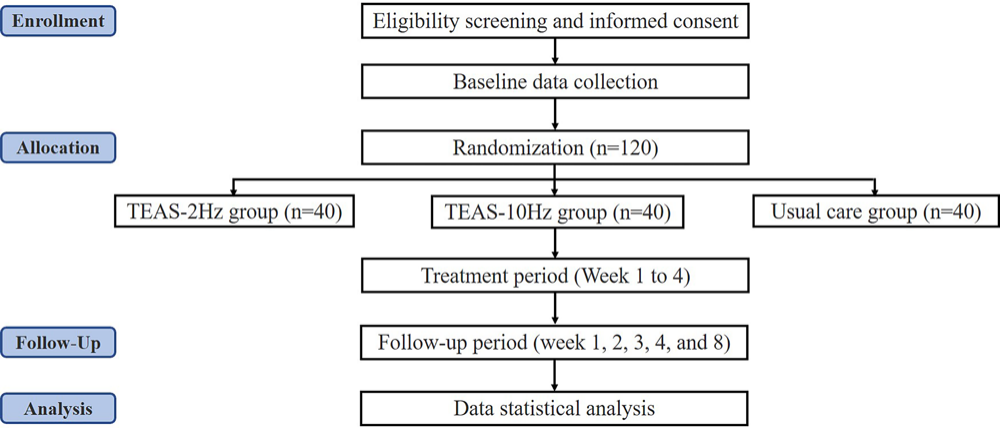
Flow chart of the trial. TEAS = transcutaneous electrical acupoint stimulation.

This study is a randomized, controlled pilot trial with 3 parallel groups. A total of 120 hypertensive patients meeting the inclusion criteria will be recruited and then randomly allocated to 3 groups of 40 patients each. All 3 groups will receive 4 weeks of usual care, and TEAS intervention groups will be additionally treated with TEAS treatment (2 Hz or 10 Hz). We will keep the stimulation location, duration, waveform, and other parameters the same for comparing the effects of different frequencies of TEAS in hypertension. The study protocol has obtained approval from the ethics committee of Beijing University of Chinese Medicine (No. 2022BZYLL1207), as well as registration at the Chinese clinical trial registry (ChiCTR2200067147). The protocol will be reported in compliance with the Standard Protocol Items (SPIRIT). The outline of the study protocol is displayed in a flow chart (Fig. 1), while the schedule of the trial process is illustrated in Table 1.

**Table 1:**
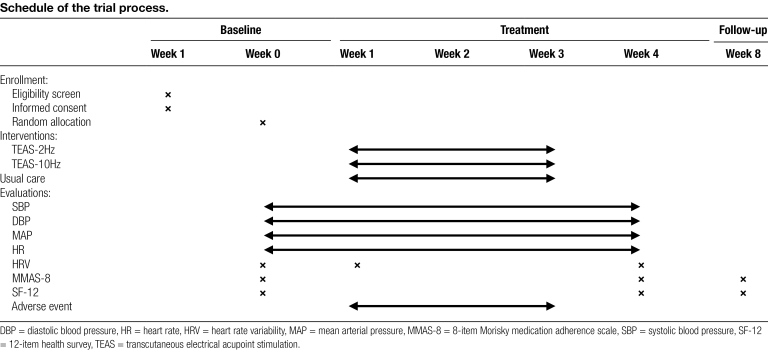
Schedule of the trial process.

### 2.2. Recruitment

The 120 participants will be screened and recruited from hospitals in Beijing. Recruitment information will be acquired by patients mainly through posters, flyers, and social media. The trained researchers will screen patients following inclusion and exclusion criteria. Patients who meet the requirement will sign informed consent before enrollment.

### 2.3. Participants

#### 2.3.1. Inclusion criteria.

1. Systolic blood pressure (SBP) of 140 to 159 mm Hg and/or diastolic blood pressure (DBP) of 90 to 99 mm Hg for individuals not taking antihypertensive drugs; SBP of 120 to 159 mm Hg and/or DBP of 80 to 99 mm Hg for individuals taking antihypertensive drugs with no change in the last month.
2. Aged 18 to 65 years old, regardless of gender.
3. No hearing and comprehension disabilities to complete the questionnaire.
4. Willing to participate in clinical observation and sign an informed consent form.

#### 2.3.2. Exclusion criteria.

1. Patients with secondary hypertension: such as acute and chronic glomerulonephritis, renal artery stenosis, gestational hypertensive syndrome, pheochromocytoma, primary aldosteronism, etc.
2. Contraindications to the use of electrical stimulation devices: with pacemakers or other implantable devices; suffering from acute illnesses, contagious diseases, and other malignant diseases; in pregnancy or lactation; breakage, inflammation, scarring, etc, at the skin of the acupuncture point; sensory impairment or allergy to electrical stimulation, etc.
3. Have taken medications other than antihypertensive drugs that may affect BP, such as glucocorticoids, in the past month.
4. Have received acupuncture or transcutaneous electrical stimulation treatment in the past year.

### 2.4. Randomization and masking

Participants enrolled in this trial will be equally allocated to the TEAS-2Hz group, TEAS-10Hz group, or usual care group by block randomization. The computer-generated random sequence will be created by an independent statistician and preserved by an investigator not involved in this trial. Every patient’s random number is to be accessed by telephone from the noninvolved investigator. Due to the trial design, participants of the usual care group will inevitably know about their grouping.

To ensure that the participants receiving TEAS and the TEAS operators will remain blinded to the allocation (2Hz or 10Hz) during the entire study, an independent investigator will set up the frequency of the stimulation device in advance and then cover the frequency display site. The distribution of the 3 groups will be hidden from the outcome evaluators and data analysts.

### 2.5. Intervention

If the participants are taking regular antihypertensive drugs, they can keep taking their medication. Every patient’s medication usage will be recorded throughout the trial by the researchers, who will also provide lifestyle education to all participants during the treatment period, such as sending information about the impact of lifestyle on BP and other related scientific knowledge.

#### 2.5.1. Usual care group.

Participants assigned to the usual care group will not be treated with TEAS interventions.

#### 2.5.2. TEAS groups.

To exploratively observe the hypotensive effect of different stimulation frequencies, this trial will set up 2 TEAS groups, in which patients will respectively receive TEAS at 2 Hz or 10 Hz, with the addition of their routine care. The interventions will last for 12 sessions of 30 minutes each, 3 sessions per week for 4 weeks. The treatment will be conducted using the TENS mode of a low frequency pulse electroacupuncture instrument (XS-998B06, Nanjing Xiaosong Medical Instrument Research Institute, Nanjing, China).

**Figure 2.**
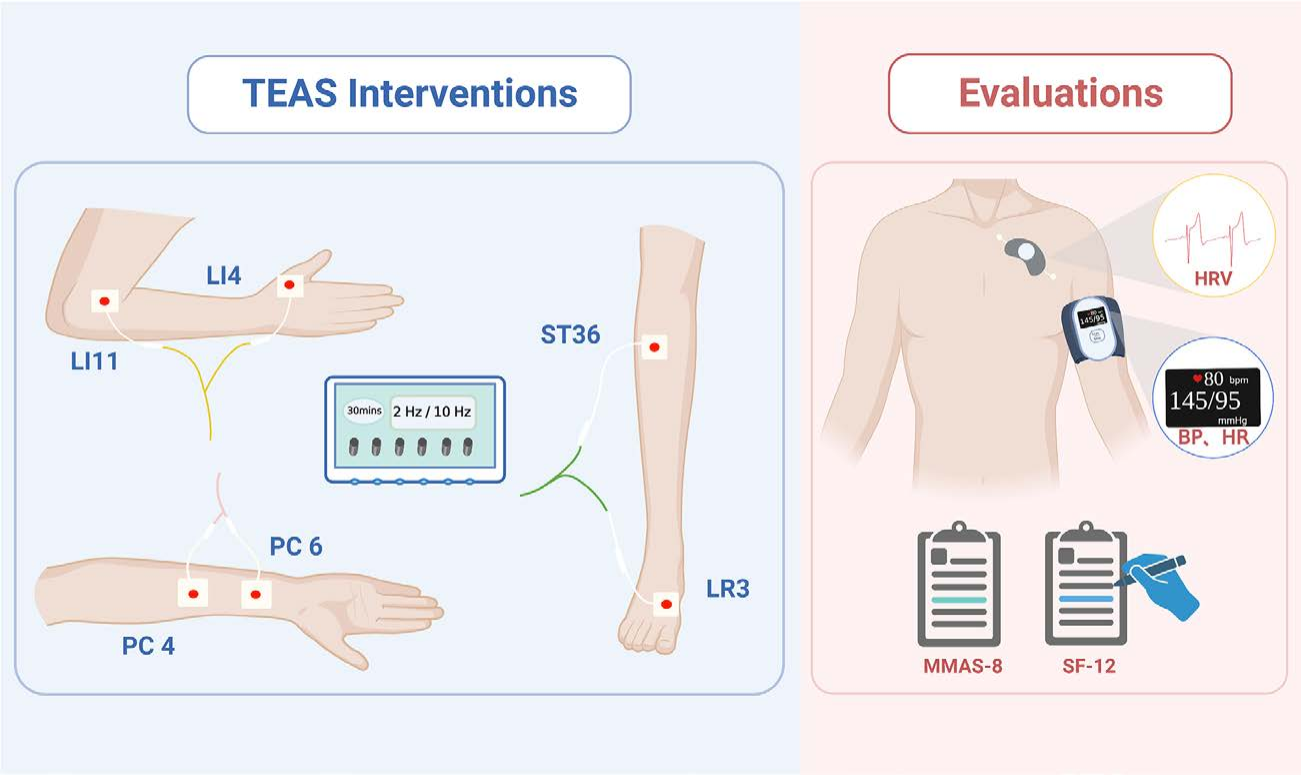
Acupoint locations (right limb as an example) and instruments for interventions and evaluations. TEAS = transcutaneous electrical acupoint stimulation; LI11 = Quchi; LI4 = Hegu; PC6 = Neiguan; PC4 = Ximen; ST36 = Zusanli; LR3 = Taichong; HRV = heart rate variability; BP = blood pressure, including systolic and diastolic blood pressure; HR = heart rate; MMAS-8 = 8-item Morisky medication adherence scale; SF-12 = 12-item health survey. Figure 2. was created on Biorender with permission to publish.

Every instrument has 6 output channels, each connected to a pair of square electrodes (40 mm × 40 mm). The patient is expected to fully expose the acupoints of the limbs in a comfortable sitting position. After cleaning the skin, bilateral acupoints will be applied by electrodes. Each pair of electrodes is connected to 3 groups of acupoints on the same side of the limb, and the acupoints are Quchi (LI11) and Hegu (LI4), Neiguan (P6) and Ximen (PC4), Zusanli (ST36) and Taichong (LR3). Figure 2 shows the location of the acupoints and the connection method with the instrument. The instrument outputs a bidirectional symmetrical narrow square wave with a pulse width of 0.4 ms-0.6 ms. The output frequency will be set at 2 Hz or 10Hz depending on the grouping of patients. The stimulation intensity of each output is adjusted to the maximum intensity tolerated by the participant and causes no pain or discomfort, and the stimulation will last for 30 minutes.

### 2.6. Primary outcome

The primary outcome will be the change in office SBP among the 3 groups from baseline to the end of the intervention (week 4). The measurement of BP will be performed with an electronic sphygmomanometer (KC2110, Shaanxi Kangshengshi Electronic Technology Co., Ltd., Shaanxi, China). Patients will be required to sit in silence for at least 5 minutes before taking their BP. Keeping the upper arm at the level of the heart, both arms will be measured at baseline and the side with the higher BP will be chosen for subsequent testing. To ensure the accuracy of the recording, the independent outcome assessor will repeat the BP measurement 3 times every 5 minutes and take the mean of the last 2 readings.

### 2.7. Secondary outcomes

Secondary outcomes will involve the change in DBP, mean arterial pressure, heart rate (HR), heart rate variability (HRV), 8-item Morisky medication adherence scale (MMAS-8), and 12-item health survey (SF-12) among the 3 groups at different time points.

The measurement of DBP and HR will be the same as SBP, at baseline, every week, and follow-up. Mean arterial pressure indicates the function of the heart and the resistance of the peripheral aorta, and it can be calculated according to this formula:^[30]^


MAP ​​ ​​ =DBP ​​ ​​ + ​​ ​​ 1/3 ​​ ​​ (SBP ​​ ​​  ​​ ​​ DBP).

Analysis of HRV can reflect the activity, homeostasis, and associated pathology of the cardiac autonomic nervous system.^[31]^ In this trial, HRV will be measured by a flexible dynamic ECG monitor (CH-E31, Zhejiang Zhi Yu Technology Co., Ltd., Zhejiang, China) at baseline, week 1, and week 4. This instrument should be attached to the left chest, about 1 finger from the clavicle, as shown in Figure 2. Before the assessment, the patient will be asked to be relaxed and quiet for at least 10 minutes and will not be verbally informed of the measurement start point, which may affect the results.

The data will be analyzed in the frequency domain from a 5-minute recording, involving low frequency (LF), high frequency (HF), LF/HF ratio, total power, very low frequency, normalized low frequency (LF norm) and normalized high frequency (HF norm).

The MMAS-8, consisting of 8 brief questions, is the most commonly used scale to investigate medication adherence in people with hypertension.^[32]^ The SF-12 is valid and equivalent for Chinese people and consists of physical component summary and mental component summary.^[33]^ Patients are advised to fill out both questionnaires at baseline, week 4, and at follow-up. Supplemental Digital Content 1, http://links.lww.com/MD/J83 shows the above 2 questionnaires.

### 2.8. Safety outcomes

Safety outcomes will be any adverse events that occur during the 4-week treatment, such as localized tingling, redness, swelling, or other adverse reactions due to electrical stimulation.

### 2.9. Sample size calculation

This pilot trial focuses on the effects of TEAS for hypertension and the optimal stimulation frequency, so as to be a preliminary for the further clinical trial. In accordance with Provisions for Drug Registration in China, the minimum number of cases (experimental group) for exploratory clinical trials is required to be 20 to 30. Browne^[34]^ considers that a sample of 30 cases is not sufficient to ensure confidentiality unless the effect size is very large. Kieser et al^[35]^ and Hertzog^[36]^ recommended 20 to 40 cases as an empirical estimate of the sample size for pretesting. Accordingly, taking full consideration of the above recommendations and the shedding rate, 40 participants per group and a total of 120 participants in 3 groups were determined.

### 2.10. Statistical analysis

The statistical software IBM SPSS Statistics (25.0, NY) will be selected for data analysis. Statistical analysis will be conducted following the principle of intention-to-treat (ITT), which involves all patients randomly assigned in the trial for ensuring the randomization principle. Missing data will be processed by the method of multiple imputation. Continuous data will be indicated as mean ± standard deviation or median and interquartile range, based on whether the normality is satisfied. As for measurements at different time points, repeated measures ANOVA and Bonferroni post hoc tests will be used for evaluation. Categorical data will be described as frequencies and percentages, and the difference among groups will be analyzed by the chi-square test or Fisher exact test. In a 2-sided test at *P* < .05, the difference will be deemed statistically significant.

### 2.11. Data management and quality control

The data measured by the sphygmomanometer and ECG monitor as well as the electronic scale scores will be uploaded in real-time to the data management terminal, which is designed for this trial by Dalian Xiran Technology Co., Ltd., Liaoning, China, with no access to all patients and treatment operators. An independent data manager will examine the accuracy and promptness of the data. All patients’ names, phone numbers, and other private information will be protected from disclosure. No data modification is allowed after the test has finished. The implementation of the trial will be reviewed every 12 months by the ethics committee of Beijing University of Chinese Medicine.

## 3. Discussion

To our knowledge, apart from evaluating the efficacy and safety of TEAS in treating hypertension, few studies have focused on the optimal stimulus parameters for TEAS treatment. The results of the trial are expected to provide evidence for TEAS treatment of hypertension and provide a reference for the research and development of related devices to optimize the stimulation parameters. Although no comparison will be made between different acupoints groups, what we concentrate more is the better efficacy of the overall regulation, which may be conducive to proactive and overall process management of BP.

### 3.1. Shedding light on the optimal stimulation parameters of TEAS is crucial for its effectiveness

Hypertension with high incidence can be fatal and disabling due to its serious complications.^[37]^ Nevertheless, the recognition and control for hypertension are insufficient, especially in patients with Grade 1 hypertension, who have a low regard for the disease and have psychological difficulties in receiving lifelong medication. TEAS is exactly such a drug-free treatment option that is more acceptable to patients. The selection of frequency plays a vital part in electrical stimulation therapy. Previous studies have shown that electrical stimulation at different frequencies acting on the periphery can facilitate specific neuropeptide releases, thus producing far-reaching physiological effects.^[38,39]^ Distinct brain regions also respond specifically to the signals of different frequencies of TENS.^[40]^ Low frequency electrical stimulation, so far, has shown a BP reduction effect in animal experiments and clinical trials.^[28,41–46]^ The frequency of electroacupuncture for hypertension is mostly 2 Hz.^[47]^ 2 Hz of electroacupuncture has been proven to inhibit sympathetic outflow and induce dilation of systemic arteries, resulting in a suppressive effect on BP.^[48]^ Se Kyun Bang et al^[24]^ screened 4 different frequencies to determine the optimal stimulation frequency of Transcutaneous median nerve stimulation for hypertension, and 10 Hz was finally chosen to be applied because of its effectiveness in lowering SBP and its comfort. In this study, we will compare the effects of TEAS at 2 Hz and 10 Hz on hypertension with the aim of exploring the optimal stimulation frequency.

### 3.2. Body-mind holistic regulation via acupoint combination may enhance the efficiency of TEAS for hypertension

Hypertension is a typical psychosomatic disease, and its occurrence and development are closely related to the degree of physical and mental alterations.^[49]^ Hypertensive patients who suffer from prolonged headaches and dizziness, or who have drug adverse effects, may experience a severe reduction of sleep quality, which will affect patients emotional state, leaving them in a state of anxiety or depression.^[50–52]^ Traditional drug therapy is characterized by a single target, making it impractical to perform multi-dimensional interventions in hypertensive patients with psychological abnormalities. TEAS can give the superiority of holistic regulation of body-mind through a precise and rational combination of acupoints. Based on the high frequency acupoints (LR3, LI4, LI11, and ST36) of acupuncture for hypertension,^[53]^ this study additionally includes Neiguan (PC6) and Ximen (PC4), which belong to the Pericardium Meridian with the stabilizing function on the mind in terms of the meridian theory. Furthermore, all the 6 acupoints located below the elbow and knee joints are both specific points, which are known as “root points” showing particular therapeutic effects for the diseases of the head, face, brain, chest, abdomen, and internal organs based on the classic acupuncture theory of “origins and junctions,” thus taking the holistic regulating effects of calming mind and lowering BP. Consequently, the acupoints combined in this study will promote the alleviation of physical symptoms and mental disorders, thereby reinforcing the antihypertensive efficacy of TEAS and enhancing the life quality of patients.

### 3.3. Potential implications of trial findings from holistic regulation with multi-perspective evaluation for future hypertension management

This study attempts to ameliorate the body-mind condition of hypertensive patients in a holistic regulation perspective, which accordingly requires multiple evaluations. In addition to BP measures, the study also assesses patients heart rates, heart rate variability, medication compliance, and quality of life. The sympathetic nervous system plays a critical function in the long-term regulation of arterial BP.^[54]^ Substantial evidence suggests that sympathetic hyperactivity occurs early in primary hypertension and involves in various stages of hypertension directly or indirectly.^[55–57]^ HR is an indicator of sympathetic excitability, and HRV reflects sympathetic and parasympathetic tension and their balance in humans.^[58]^ The results of the above 2 indicators may explain the mechanism of TEAS to reduce BP from a certain perspective, and also contribute to managing hypertension in patients with heart disease, especially those who are resistant to medication or have side effects. Additionally, the management of hypertension is also closely related to medication adherence and the quality of life of patients. MMAS-8 will be used to learn about the medication habits of the enrolled patients and SF-12 provides an overall assessment of both physical and psychological aspects with brief questions. Hypertension requires combined modality therapy and long-term management, and taking medication over a long time not only burdens the economy but also imposes side effects. TEAS being both safe and effective, economical and well adhered to, makes it feasible for patients to manage their BP proactively and persistently. Therefore, the findings of this trial may potentially offer evidence that TEAS might contribute to the realization of active and whole-course BP management.

## 4. Limitations

This study has some limitations. First, we are unable to blind all patients. No sham TEAS interventions will be used in the usual care group, which causes this group unable to be blind to their grouping, so there may be some psychological factors that affect the BP of patients in this group. While the objective of this study is to screen for optimal stimulation parameters of TEAS, we can try to ensure that patients in the 2 TEAS intervention groups are blind to the specific stimulation frequency. Besides, BP is measured and recorded using automated equipment to minimize bias in essential results. Second, although 4 weeks is the common clinical treatment period for acupuncture, future studies may require a longer treatment period and follow-up time to investigate the profound effects of TEAS on BP. Finally, further expansion of the sample size in future studies is necessary to confirm the findings of this trials.

## Acknowledgments

All authors would like to appreciate Dalian Xiran Technology Co., Ltd. for cooperating with our research team and for facilitating us to better manage the data.

## Author contributions

**Conceptualization:** Ling-Hui Ma, Zhou Zhang, Liang-Xiao Ma.

**Formal analysis:** Xu Qian, Tian-Yi Sun.

**Funding acquisition:** Liang-Xiao Ma.

**Methodology:** Ling-Hui Ma, Zhou Zhang, Jie-Dan Mu, Xu Qian, Qin-Yong Zhang.

**Project administration:** Jie-Dan Mu.

**Writing – original draft:** Ling-Hui Ma, Zhou Zhang.

**Writing – review & editing:** Liang-Xiao Ma.

## Supplementary Material


