# Supplementary material for: Biofeedback physical regulation of hypertension based on acupoints: A clinical trial
Source: Medicine (Baltimore). 2023 Jun 23;102(25):e33946. doi: 10.1097/MD.0000000000033946 (PMC10289684; doi:10.1097/MD.0000000000033946)
Supplement: Supplementary file 1 [file medi-102-e33946-s001.pdf]

English version of questionnaires：

The 8-item Morisky Medication Adherence Scale (MMAS-8)

Below is a list of statements. Please indicate the extent to which you agree or disagree with them by marking the number of the appropriate box. There are no right or wrong answers. Please give honest answers; otherwise the result would not be valid.

1. Do you sometimes forget to take your antihypertensive medication?
(1) Yes
(2) No

2.Over the past 2 weeks, were there any days when you did not take your antihypertensive medication?
(1) Yes
(2) No

3. Have you ever cut back or stopped taking your antihypertensive medication without telling your doctor because you felt worse when you took it?
(1) Yes
(2) No

4. When you travel or leave home, do you sometimes forget to bring your antihypertensive medication?
(1) Yes
(2) No

5. Did you take your antihypertensive medicine yesterday?
(1) Yes
(2) No

6. When you feel like your blood pressure are under control, do you sometimes stop taking your antihypertensive medication?
(1) Yes
(2) No

7. Do you ever feel hassled about sticking to your treatment plan?
(1) Yes
(2) No

8. How often do you have difficulty remembering to take all your antihypertensive medication?
(1) Never/Rarely
(2) Once in a while

(3) Sometimes
(4) Usually
(5) All the time

SF-12 health survey

Instructions: This questionnaire asks for your views about your health. This information will help keep track of how you feel and how well you are able to do your usual activities.

Please answer every question by marking one box. If you are unsure about how to answer, please give the best answer you can.

1. In general, would you say your health is:
(1) Excellent
(2) Very Good
(3) Good
(4) Fair
(5) Poor

2. Does your health now limit you in moderate activities, such as moving a table, pushing a vacuum cleaner, bowling, or playing golf? If so, how much?
(1) Yes, limited a lot
(2) Yes, limited a little
(3) No, not limited at all

3. Does your health now limit you in Climbing several flights of stairs? If so, how much?
(1) Yes, limited a lot
(2) Yes, limited a little
(3) No, not limited at all

4.During the past 4 weeks, have you accomplished less than you would like with your work or other regular daily activities as a result of your physical health?
(1) YES
(2) NO

5. During the past 4 weeks, have you been limited in the kind of work or other activities as a result of your physical health?
(1) YES
(2) NO

6. During the past 4 weeks, have you accomplished less than you would like with your work or other regular daily activities as a result of any emotional problems (such as feeling depressed or anxious)?
(1) YES
(2) NO

7. During the past 4 weeks, have you not done work or other activities as carefully as usual as a result of any emotional problems (such as feeling depressed or anxious)?
(1) YES
(2) NO

8. During the past 4 weeks, how much did pain interfere with your normal work (including both work outside the home and housework)?
(1) Not at all
(2) A little bit
(3) Moderately
(4) Quite a bit
(5) Extremely

These questions are about how you feel and how things have been with you during the past 4 weeks. For each question, please give the one answer that comes closest to the way you have been feeling.

9. How much of the time during the past 4 weeks have you felt calm and peaceful?
(1) All of the time
(2) Most of the time
(3) A good bit of the time
(4) Some of the time
(5) A little of the time
(6) None of the time

10. How much of the time during the past 4 weeks did you have a lot of energy?
(1) All of the time
(2) Most of the time
(3) A good bit of the time
(4) Some of the time
(5) A little of the time
(6) None of the time

11. How much of the time during the past 4 weeks have you felt downhearted and blue?
(1) All of the time
(2) Most of the time
(3) A good bit of the time

(4) Some of the time
(5) A little of the time
(6) None of the time

12. During the past 4 weeks, how much of the time has your physical health or emotional problems interfered with your social activities (like visiting with friends, relatives, etc.)?
(1) All of the time
(2) Most of the time
(3) Some of the time
(4) A little of the time
(5) None of the time

中文版问卷

Morisky 服药依从性量表(MMAS-8)

下面有一些陈述，请根据这些陈述与您的符合程度在适当的数字上打勾。答案没有对错之分，请您尽量诚实地回答问卷，否则结果是无效的。

1.您是否有时忘记服降压药?
(1)是
(2)否

2.过去 2 周内，是否有一天或几天您忘记服降压药?
(1)是
(2)否

3.治疗期间，当您觉得症状加重或者出现其他症状时，您是否未告知医生而自行减少药量或停止服降压药?
(1)是
(2)否

4.当您外出旅行或长时间离家时，您是否有时忘记随身携带降压药物?
(1)是
(2)否

5.昨天您服降压药了吗?
(1)是
(2)否

6.当您觉得自己的血压已经得到控制时，您是否停止过服降压药?
(1)是
(2)否

7.您是否觉得要坚持治疗计划有困难?
(1)是
(2)否

8.您觉得要记住按时按量服降压药很困难吗?
(1)从不
(2)偶尔
(3)有时
(4)经常
(5)所有时间

健康调查 12 项简表（SF-12）

说明:这份问卷调查的目的是希望通过多方面的问题内容，包括整体健康、体能、日常活动精力、身体疼痛、心理健康和社交活动等，加深我们对您健康状况及日常生活质量的了解，所以请您尽量回答问卷内的所有问题，并且选出最合适的答案，每一个问题只可选择一个答案。如对某一问题不能肯定或不太清楚，就选出最近似的一个答案。

Q1. 总体来说，您认为您的健康状况如何？

(1)非常好

(2)很好

(3)好

(4)一般

(5)差

Q2.以您目前的健康状况，进行中等强度的活动，例如搬桌子、使用吸尘器清洁地面、玩保龄球或打太极拳，有没有受到限制?如果有，程度如何?

(1) 有很大限制

(2) 有一点限制

(3)没有任何限制

Q3. 您目前的健康状况是否影响您步行上楼?

(1) 有很大限制

(2) 有一点限制

(3)没有任何限制

Q4.在过去的 4 个星期里，您是否会因为身体健康的原因，在日常生活或工作中感到力不从心?

(1)会

(2)不会

Q5.在过去 4 个星期的工作或日常活动中，您是否会因为身体健康的原因而令您的工作或活动受到限制?

(1)会

(2)不会

Q6. 在过去的 4 个星期里，您是否会因为情绪方面的原因(比如感到沮丧或焦虑)而令您在工作或日常活动中感到力不从心?

(1)会

(2)不会

Q7.在过去 4 个星期的工作或日常活动中，您会否因为情绪方面的原因(比如感到沮丧或者焦虑)而令您的工作或活动受到限制?

(1)会

(2)不会

Q8.在过去 4 个星期里，您身体上的疼痛对您的日常工作(包括上班和家务)有多大影响?

(1)完全没有影响

(2)有很少影响

(3)有一些影响

(4)有较大影响

(5)有非常大的影响

以下问题是关于您在过去 4 个星期里自我感觉及其他的情况。针对每一个问题，请选择一个最接近您感觉的答案。

Q9. 在过去 4 个星期里，您有多少时间感到心平气和?

(1) 所有时间

(2) 大部分时间

(3) 很多时间

(4) 有时

(5) 很少时间

(6) 从来没有

Q10.在过去 4 个星期里，您有多少时间感到精力充足?

(1) 所有时间

(2) 大部分时间

(3) 很多时间

(4) 有时

(5) 很少时间

(6) 从来没有

Q11.在过去 4 个星期里，您有多少时间觉得心情不好、闷闷不乐或沮丧?

(1) 所有时间

(2) 大部分时间

(3) 很多时间

(4) 有时

(5) 很少时间

(6) 从来没有

Q12.在过去 4 个星期里，有多少时间由于您身体健康或情绪问题而妨碍您的社交活动(比如探亲、访友等)?

(1) 所有时间

(2) 大部分时间

(3) 有时

(4) 很少时间

(5) 从来没有
